# Vaccine effectiveness against COVID‐19 among symptomatic persons aged ≥12 years with reported contact with COVID‐19 cases, February–September 2021

**DOI:** 10.1111/irv.12973

**Published:** 2022-02-15

**Authors:** Jessie R. Chung, Sara S. Kim, Edward A. Belongia, Huong Q. McLean, Jennifer P. King, Mary Patricia Nowalk, Richard K. Zimmerman, Krissy Moehling Geffel, Emily T. Martin, Arnold S. Monto, Lois E. Lamerato, Manjusha Gaglani, Eric Hoffman, Marcus Volz, Michael L. Jackson, Lisa A. Jackson, Manish M. Patel, Brendan Flannery

**Affiliations:** ^1^ Centers for Disease Control and Prevention Atlanta Georgia USA; ^2^ Marshfield Clinic Research Institute Marshfield Wisconsin USA; ^3^ University of Pittsburgh Schools of the Health Sciences and University of Pittsburgh Medical Center Pittsburgh Pennsylvania USA; ^4^ School of Public Health University of Michigan Ann Arbor Michigan USA; ^5^ Henry Ford Health System Detroit Michigan USA; ^6^ Baylor Scott and White Health Dallas TX USA; ^7^ Texas A&M University College of Medicine Temple Texas USA; ^8^ Kaiser Permanente Washington Health Research Institute Seattle Washington USA

**Keywords:** COVID‐19, SARS‐CoV‐2, vaccine effectiveness

## Abstract

**Background:**

Individuals in contact with persons with COVID‐19 are at high risk of developing COVID‐19; protection offered by COVID‐19 vaccines in the context of known exposure is poorly understood.

**Methods:**

Symptomatic outpatients aged ≥12 years reporting acute onset of COVID‐19‐like illness and tested for SARS‐CoV‐2 between February 1 and September 30, 2021 were enrolled. Participants were stratified by self‐report of having known contact with a COVID‐19 case in the 14 days prior to illness onset. Vaccine effectiveness was evaluated using the test‐negative study design and multivariable logistic regression.

**Results:**

Among 2229 participants, 283/451 (63%) of those reporting contact and 331/1778 (19%) without known contact tested SARS‐CoV‐2‐positive. Adjusted vaccine effectiveness was 71% (95% confidence interval [CI], 49%–83%) among fully vaccinated participants reporting a known contact versus 80% (95% CI, 72%–86%) among those with no known contact (*p*‐value for interaction = 0.2).

**Conclusions:**

This study contributes to growing evidence of the benefits of vaccinations in preventing COVID‐19 and support vaccination recommendations and the importance of efforts to increase vaccination coverage.

## 1 INTRODUCTION

Individuals in contact with persons with COVID‐19 disease are at high risk of SARS‐CoV‐2 infection and developing COVID‐19 themselves. The US Centers for Disease Control and Prevention (CDC) recommends vaccination as the best tool to prevent COVID‐19 among persons aged ≥5 years in conjunction with non‐pharmaceutical interventions (NPIs) such as hand washing, mask wearing, and physical distancing. Repeated, extended exposures in proximity to persons with SARS‐CoV‐2 can increase the risk of becoming infected.^1^ Having close contact with a person with COVID‐19, such as within a household, is a main source of new SARS‐CoV‐2 infections. Thus, CDC recommends persons who are not yet fully vaccinated to seek testing immediately after finding out they have had close contact, even if they do not have symptoms.

Data are limited regarding how COVID‐19 vaccine effectiveness (VE) may vary by intensity of exposure.^2^ One approach to assess impact of exposure would be to evaluate VE against symptomatic illness among individuals with known contact, compared with individuals unaware of close exposure. These data may contribute to efforts to increase COVID‐19 vaccine uptake among persons who have not yet received vaccines and possibly inform NPI strategies as coverage increases [2]. In this report, we build upon prior studies of COVID‐19 VE from the US Influenza Vaccine Effectiveness (Flu VE) Network to present VE against symptomatic laboratory‐confirmed SARS‐CoV‐2 infection among persons aged ≥12 years with and without known contact with a person with SARS‐CoV‐2 infection from February to September 2021.

## 2 METHODS

Methods used for estimating VE against laboratory‐confirmed symptomatic SARS‐CoV‐2 infection or COVID‐19 among persons seeking medical care/testing for SARS‐CoV‐2 in Flu VE Network study sites have been previously described.^3^ Briefly, research staff screened persons who sought outpatient medical care (i.e., telehealth, primary care, urgent care, and/or emergency department) or clinical SARS‐CoV‐2 testing using a standard case‐definition for COVID‐like illness that included onset of fever/feverishness, cough, or loss of taste/smell with symptom duration <10 days.^4^ Research staff contacted potentially eligible outpatients in person, by telephone, or email to confirm eligibility and enroll participants who consented verbally or in writing. Standardized questionnaires collected demographic information, healthcare‐related occupation, and COVID‐19 vaccination. Participants were also asked “Did you have contact with a lab‐confirmed COVID‐19 case in the 14 days before your symptoms started?”. Participants who answered “yes” were categorized as having “known contact,” and participants who answered “no” were categorized as having “no known contact.” Participants had SARS‐CoV‐2 molecular testing on respiratory specimens collected within 10 days of illness onset; results were used to classify SARS‐CoV‐2‐positive cases and test‐negative controls.

For this analysis, we included participants aged ≥12 years with illness onset between February 1 and September 30, 2021. Beginning dates of inclusion in analyses varied by site according to local COVID‐19 vaccination policies for all persons by age group as vaccines became available (February 1 for persons aged ≥65 years, March 22 for persons aged 16–64 years, and May 12 for persons aged 12–15 years). We determined vaccination status through participant interviews, and verified vaccination based on participant‐provided vaccination record cards, and/or documentation of vaccination in electronic medical or state immunization information systems. Fully vaccinated participants were defined as those who received two doses of mRNA vaccine (Pfizer‐BioNTech BNT162b2 or Moderna mRNA‐1273) or one dose of Johnson and Johnson/Janssen vaccine (JNJ‐784367350) ≥ 14 days before illness onset.^5^ Partially vaccinated participants were defined as those who received at least one dose of mRNA vaccine ≥14 days before illness onset but who were not fully vaccinated. Those who had no documentation of COVID‐19 vaccination prior to illness onset were defined as unvaccinated. Participants whose first dose was received <14 days prior to illness onset (*n* = 141) were excluded from VE analyses.

We used the test‐negative design to evaluate VE of currently available SARS‐CoV‐2 vaccines against symptomatic, laboratory‐confirmed outpatient COVID‐19^6^ among participants reporting contact with persons with confirmed COVID‐19 and those reporting no known contact. Persons who did not complete the question regarding known contact were excluded from primary analyses. VE was calculated as 1—adjusted odds ratio of vaccination among symptomatic SARS‐CoV‐2‐test‐positive participants versus symptomatic test‐negative participants (controls) using multivariable logistic regression. An interaction term for known contact and vaccination status was assessed. Models were adjusted as previously described and included age, study site, enrollment period, and self‐reported race/ethnicity.^3^ We performed stratified analyses by (1) time of illness onset using February–May 2021 as a pre‐Delta variant period and July—September 2021 as the Delta variant‐predominant period and (2) by age group (as 12–49 years and >49 years due to sample size).^7^

We performed several sensitivity analyses. We compared VE using plausible self‐report of vaccination to VE using only documented vaccination status, where plausibility was determined by ability to report credible location of vaccination, as previously described.^3^ We also compared findings when persons who identified as working in healthcare were excluded and also when persons with unknown status were classified as having had no known contact. Statistical analyses were conducted using SAS version 9.4 (SAS Institute Inc., Cary, NC, USA). This research activity involving human subjects was reviewed by the Institutional Review Boards (IRB) of the CDC and Baylor Scott and White Health, Marshfield Clinic Research Institute, University of Michigan, Henry Ford Health System, and University of Pittsburgh and was conducted consistent with applicable federal law and CDC policy (See 45 C.F.R. part 46; 21 C.F.R. part 56). The Kaiser Permanente Washington Health Research Institute IRB determined that this activity was research not involving human subjects under a nondisclosure agreement with CDC.

## 3 RESULTS

Of 3384 symptomatic persons aged ≥12 years enrolled from February 1 to September 30, 2021, information on contact with a person with laboratory‐confirmed COVID‐19 during the 14 days before illness onset was available from 2229 (65.9%) participants: 451 (20%) reported contact and 1778 (80%) reported no known contact. Among participants reporting contact, 283 (63%) were SARS‐CoV‐2 test‐positive cases compared with 331 (19%) of participants without known contact. Participants reporting contact with persons with COVID‐19 were more likely than those with unknown contact to be healthcare workers, aged <65 years, household contacts of children aged <12 years, tested for SARS‐CoV‐2 less than 4 days since illness onset, and have no documented COVID‐19 vaccination (Table 1).

**TABLE 1:** Characteristics of enrolled participants by self‐reported known contact with a person with SARS‐CoV‐2 in the 14 days prior to illness onset, US Flu VE Network, February 1 to September 30, 2021

		Known contact		No known contact		
	Total	*n*	Column %	*n*	Column %	*p*‐value^a^
**Total**	2229	451	100	1778	100	
**Age group (years)**						<0.01
12–15	99	18	4	81	5	
16–49	1182	267	59	915	51	
50–64	523	112	25	411	23	
≥65	425	54	12	371	21	
**Study site**						<0.01
Michigan	312	102	23	210	12	
Pennsylvania	358	75	17	283	16	
Texas	377	102	23	275	15	
Washington	740	90	20	650	37	
Wisconsin	442	82	18	360	20	
**Sex**						0.45
Female	1438	298	66	1140	64	
Male	790	153	34	637	36	
**Race/ethnicity**						0.07
Black non‐Hispanic	186	33	7	153	9	
Hispanic	159	38	9	121	7	
Other non‐Hispanic	211	30	7	181	10	
White non‐Hispanic	1661	346	77	1315	74	
**Underlying condition** ^b^						0.12
No	1402	270	61	1132	65	
Yes	789	174	39	615	35	
**Child aged <12 years living in household**						<0.01
None	1589	286	63	1303	73	
≥1	640	165	37	475	27	
**Healthcare worker** ^c^						<0.01
No	1770	329	79	1441	88	
Yes	275	87	21	188	12	
**Documented 2020–2021 influenza vaccination**						<0.01
No	1606	368	82	1238	70	
Yes	623	83	18	540	30	
**Prior SARS‐CoV‐2 infection** ^d^						0.87
No	1993	405	91	1588	91	
Yes	192	40	9	152	9	
**Symptom onset to specimen collection interval (days)**						<0.01
0–3	1134	258	57	876	49	
4–6	771	128	28	643	36	
7–10	324	65	14	259	15	
**COVID‐19 vaccination status** ^e^						<0.01
Fully vaccinated	1134	183	43	951	56	
Partially vaccinated	154	26	6	128	8	
Unvaccinated	828	220	51	608	36	
**SARS‐CoV‐2 status**						<0.01
Negative	1615	168	37	1447	81	
Positive	614	283	63	331	19	

Abbreviation: VE, vaccine effectiveness.

^a^ *P*‐value is for the chi‐square test where *p*<0.05 was considered statistically significant.

^b^ Underlying condition (e.g., heart disease, lung disease, diabetes, cancer, liver or kidney disease, immune suppression, or high blood pressure) is self‐reported; 38 participants (7 with known contact and 31 participants with no known contact) missing underlying condition status.

^c^ Work in healthcare setting is self‐reported; 21 participants missing healthcare worker status; 163 participants <18 years old were not asked this question.

^d^ Prior SARS‐CoV‐2 infection is self‐reported; 44 participants (6 with known contact and 44 with no known contact) missing prior infection status.

^e^ There were 112 participants who had indeterminate vaccination status (22 with known contact and 90 with no known contact).

Among participants reporting known contact, 183 (43%) were fully vaccinated ≥14 days before illness onset versus 951 (56%) of those without known contact (Table 1). Most of the 1288 vaccinated participants received mRNA vaccines (1204, 93%); 761(59%) received Pfizer‐BioNTech, and 443 (34%) received Moderna. Few participants (81, 6%) received Johnson and Johnson/Janssen vaccine or a vaccine of unknown type (3, <1%). Over 90% of vaccinated participants were vaccinated prior to mid‐May 2021. The median number of days between most recent dose and onset of symptoms was 113 days (interquartile range 67–155) with no differences observed by the product received.

**FIGURE 1:**
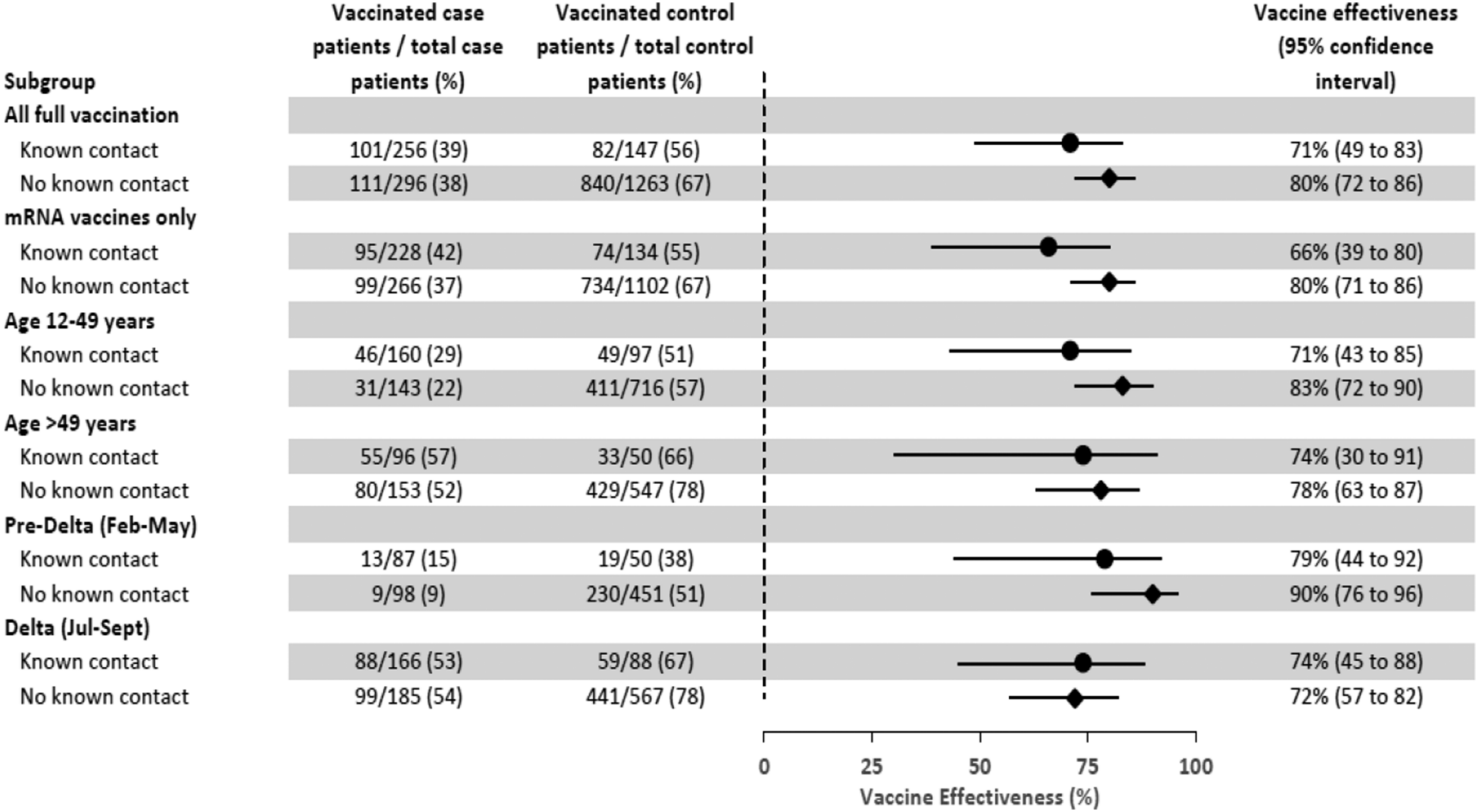
Estimates of vaccine effectiveness^a^ against laboratory‐confirmed symptomatic COVID‐19 among participants with and without reported known contact with persons with COVID‐19 during the 14 days before illness onset, US Flu VE Network, February 1 to September 30, 2021. CI, confidence interval. ^a^Vaccine effectiveness (VE) of full vaccination by documented records versus unvaccinated. Model adjusted for study site, age in years (continuous), enrollment period (natural cubic spline with 3 percentile knots of interval between January 1, 2021 and illness onset date), and self‐reported race/ethnicity

Among all participants with non‐indeterminate vaccination status (*n* = 2116), VE of documented full vaccination against laboratory‐confirmed, symptomatic COVID‐19 with any COVID‐19 vaccine was 72% (95% confidence interval [CI]: 64–78). Similar estimates were observed among participants with and without known contact (Figure 1). Adjusted VE among fully vaccinated participants with known contact was 71% (95% CI: 49–83) compared with 80% (95% CI: 72–86) among fully vaccinated participants with no known contact (*p*‐value for interaction = 0.2). Significant interaction was not observed for any comparison (*p*‐value ≥0.1 for all). In sensitivity analyses, overall VE was similar when known healthcare workers were excluded, participants with unknown contact (*n* = 1155) were classified as “no known contact,” plausible self‐reported doses were incorporated, and partially vaccinated participants were included (supporting information Table S1).

VE point estimates of full vaccination against symptomatic laboratory‐confirmed COVID‐19 among all participants regardless of reported exposure status differed by mRNA product (Moderna VE 81% [95% CI: 73–86], Pfizer VE 66% [95% CI: 56–73]), age group (70% [95% CI: 59–78] among participants aged 16–49 years, 80% [95% CI: 68–88] among participants 50–64 years, and 61% [95% CI: 29–79] among participants aged ≥65 years), and enrollment period (86% [95% CI 77–92] for participants with onset in the pre‐Delta‐variant period and 59% [95% CI: 45–69] for participants with onset during Delta variant‐predominance period).

## 4 DISCUSSION

Vaccination against COVID‐19 disease provided protection against laboratory‐confirmed, symptomatic outpatient illness among individuals who reported known contact with a person with COVID‐19. Participants who reported known contact with a person with COVID‐19 were more likely to test positive for SARS‐CoV‐2 compared with participants who reported no known contact. Those who reported a known contact were more likely to report living in a household with at least one child aged <12 years or to report work in a healthcare setting; our findings did not differ substantially when persons who work in a healthcare setting were excluded. In addition, we did not detect a statistically significant difference by known contact status when participants were further stratified by illness onset into pre‐Delta variant versus Delta variant circulation periods.

Having contact with a known person with SARS‐CoV‐2 infection substantially increases the likelihood of testing positive for SARS‐CoV‐2. Contact tracing and transmission studies suggest that household settings have the highest secondary attack rates, with an estimated pooled secondary transmission rate of 21.1% (95% CI: 17.4–24.8).^1^ Although we were unable to categorize the setting of known exposure in our study, other studies have compared secondary transmission from a household contact compared with other forms of contact and highlighted the importance of household transmission compared with occupational, social, or transportation exposures.^8^, ^9^ The importance of household transmission is likely due to prolonged exposures in proximity with fewer protective measures in place. In one study, unvaccinated or partially vaccinated persons were more likely to transmit SARS‐CoV‐2 virus compared with fully vaccinated persons.^9^ We show equivalent, high levels of protection of full vaccination against symptomatic, laboratory‐confirmed COVID‐19 regardless of known contact.

We build upon prior published findings from the Flu VE Network in several ways. This analysis includes four additional months of data compared with an earlier evaluation of COVID‐19 VE between February and May 2021.^3^ Despite predominance of the Delta variant in the latter study period,^7^ our findings show protection against laboratory‐confirmed symptomatic illness. A decline in VE point estimates in the latter period could be attributed to reduced protection against the Delta or a result of waning protection of the initial vaccination series.^10^, ^11^ However, this study was underpowered to evaluate and disentangle these factors. Similar to other published reports, we detected lower adjusted VE of Pfizer‐BioNTech vaccine compared with Moderna vaccine.^12^ We summarize VE of full vaccination against laboratory‐confirmed symptomatic outpatient illness in the period of time prior to when third/booster doses became available and were recommended, as well as the period of time when vaccination was recommended for US children aged 5 to 12 years of age.^13^, ^14^ Booster doses of all COVID‐19 vaccines are currently available for adults aged ≥18 years in the USA.^15^

Our study was subject to at least five limitations. First, we did not assess the nature of reported contact and do not have information about whether the exposure was a household member, occupational, or other type. Second, we did not collect information about timing of reported known contact within the 14 days prior to illness onset. Participants could have been infected prior to reported exposure. Third, some participants might have been aware of their SARS‐CoV‐2 test status when they completed the enrollment questionnaire, which could have influenced responses to the known contact question.^8^ While the test‐negative design reduces bias due to differences in healthcare‐seeking behavior among vaccinated and unvaccinated persons,^6^ vaccinated cases could have been more motivated to participate in this study. Fourth, we did not ask about NPIs, duration, or distance of contact of the known exposure. Differences in exposures and prevention measures among vaccinated and unvaccinated participants could have been associated with likelihood of testing positive for SARS‐CoV‐2 infection. Finally, our study was unable to account for differences in timing of vaccination that could result in an observed waning of effectiveness with increased time since vaccination.

This study contributes to growing evidence of COVID‐19 VE against symptomatic illness, among members of the general population who have contact with persons with COVID‐19. These findings support recommendations for COVID‐19 vaccination for the prevention of symptomatic illness and highlight the importance of continued efforts to increase vaccination coverage.

## FUNDING INFORMATION

This work was supported by the US Centers for Disease Control and Prevention through cooperative agreements U01IP001034‐U01IP001039. At Pittsburgh, the project was also supported by the National Institutes of Health through grant UL1TR001857.

## DISCLAIMERS

The findings and conclusions in this report are those of the authors and do not necessarily represent the official position of the Centers for Disease Control and Prevention/Agency for Toxic Substances and Disease Registry. Vaccination data from Pennsylvania were supplied by the Bureau of Health Statistics and Registries, Pennsylvania Department of Health, Harrisburg, Pennsylvania. The Pennsylvania Department of Health specifically disclaims responsibility for any analyses, interpretations, or conclusions.

## AUTHOR CONTRIBUTIONS

**Jessie Chung:** Conceptualization; formal analysis; investigation; project administration; visualization. **Sara Kim:** Conceptualization; formal analysis; investigation; project administration; visualization. **Edward Belongia:** Conceptualization; funding acquisition; investigation; methodology; project administration; resources; supervision; visualization. **Huong Q. McLean:** Conceptualization; funding acquisition; investigation; methodology; project administration; resources; supervision; visualization. **Jennifer King:** Conceptualization; funding acquisition; investigation; project administration; resources; supervision; visualization. **Mary Patricia Nowalk:** Conceptualization; funding acquisition; investigation; methodology; project administration; resources; supervision; visualization. **Richard Zimmerman:** Conceptualization; funding acquisition; investigation; methodology; project administration; resources; supervision; visualization. **Krissy Moehling:** Conceptualization; funding acquisition; investigation; methodology; project administration; resources; supervision; visualization. **Emily Martin:** Conceptualization; funding acquisition; investigation; methodology; project administration; resources; supervision; visualization. **Arnold Monto:** Conceptualization; funding acquisition; investigation; methodology; project administration; resources; supervision; visualization. **Lois Lamerato:** Conceptualization; funding acquisition; investigation; methodology; project administration; resources; supervision; visualization. **Manjusha Gaglani:** Conceptualization; funding acquisition; investigation; methodology; project administration; resources; supervision; visualization. **Eric Hoffman:** Conceptualization; investigation; project administration; resources; visualization. **Marcus Volz:** Conceptualization; investigation; project administration; resources; visualization. **Michael Jackson:** Conceptualization; funding acquisition; investigation; methodology; project administration; resources; supervision; visualization. **Lisa Jackson:** Conceptualization; funding acquisition; investigation; methodology; project administration; resources; supervision; visualization. **Manish Patel:** Conceptualization; funding acquisition; investigation; methodology; project administration; resources; supervision; visualization. **Brendan Flannery:** Conceptualization; funding acquisition; investigation; methodology; project administration; resources; supervision; visualization.

### PEER REVIEW

The peer review history for this article is available at https://publons.com/publon/10.1111/irv.12973.

## Supporting information


**Supplemental Table S1.** Results of sensitivity analyses of vaccine effectiveness against laboratory‐confirmed symptomatic COVID‐19, US Flu VE Network, February 1–September 30, 2021

## Data Availability

Requests for access to data may be made to the corresponding author.
