# Supplementary material for: Vaccine effectiveness against COVID‐19 among symptomatic persons aged ≥12 years with reported contact with COVID‐19 cases, February–September 2021
Source: Influenza Other Respir Viruses. 2022 Feb 15;16(4):673–9. doi: 10.1111/irv.12973 (PMC9111783; doi:10.1111/irv.12973)
Supplement: Supplementary file 1 — Supplemental Table S1. Results of sensitivity analyses of vaccine effectiveness against laboratory‐confirmed symptomatic COVID‐19, US Flu VE Network, February 1–September 30, 2021 [file IRV-16-673-s001.docx]

**Supplemental Table**. Results of sensitivity analyses of vaccine effectiveness against laboratory-confirmed symptomatic COVID-19, US Flu VE Network, February 1–September 30, 2021

|  | **SARS-CoV-2-Positive CLI (Cases)** | | | **SARS-CoV-2-Negative CLI Controls** | | | **Adjusted* VE** | |
| --- | --- | --- | --- | --- | --- | --- | --- | --- |
|  | # Vaccinated | Total | % Vaccinated | # Vaccinated | Total | % Vaccinated | VE % | (95% CI) |
| **Include partially vaccinated participants** |  |  |  |  |  |  |  |  |
| Known contact | 117 | 268 | 44 | 98 | 161 | 61 | 73 | (54 to 84) |
| No known contact | 133 | 309 | 43 | 960 | 1355 | 71 | 78 | (69 to 84) |
| **Exclude healthcare workers** |  |  |  |  |  |  |  |  |
| Known contact | 76 | 215 | 35 | 58 | 110 | 53 | 73 | (47 to 86) |
| No known contact | 101 | 267 | 38 | 738 | 1116 | 66 | 80 | (70 to 86) |
| **Assume persons with unknown status had no known contact** |  |  |  |  |  |  |  |  |
| No known contact | 230 | 542 | 42 | 977 | 1486 | 66 | 71 | (62 to 78) |
| **Include plausible self-reported doses** |  |  |  |  |  |  |  |  |
| Known contact | 104 | 255 | 41 | 84 | 147 | 57 | 72 | (51 to 84) |
| No known contact | 117 | 293 | 40 | 846 | 1241 | 68 | 80 | (71 to 86) |

CLI = COVID-19-like illness

*Vaccine effectiveness (VE) adjusted for study site, age in years (continuous), enrollment period (natural cubic spline with 3 percentile knots of interval between January 1, 2021 and illness onset date), and self-reported race/ethnicity.
